# Genome Assembly of the Nematode *Rhabditoides Inermis* From a Complex Microbial Community

**DOI:** 10.1093/gbe/evae230

**Published:** 2024-11-07

**Authors:** Christian Rödelsperger, Waltraud Röseler, Marina Athanasouli, Sara Wighard, Matthias Herrmann, Ralf J Sommer

**Affiliations:** Department for Integrative Evolutionary Biology, Max Planck Institute for Biology, Tübingen 72076, Germany; Department for Integrative Evolutionary Biology, Max Planck Institute for Biology, Tübingen 72076, Germany; Department for Integrative Evolutionary Biology, Max Planck Institute for Biology, Tübingen 72076, Germany; Department for Integrative Evolutionary Biology, Max Planck Institute for Biology, Tübingen 72076, Germany; Department for Integrative Evolutionary Biology, Max Planck Institute for Biology, Tübingen 72076, Germany; Department for Integrative Evolutionary Biology, Max Planck Institute for Biology, Tübingen 72076, Germany

**Keywords:** comparative genomics, metagenome, *Pristionchus pacificus*, *Caenorhabditis elegans*, *Vanrija albida*, *Nicrophorus vespilloides*

## Abstract

Free-living nematodes such as *Caenorhabditis elegans* and *Pristionchus pacificus* are powerful model systems for linking specific traits to their underlying genetic basis. To trace the evolutionary history of specific traits or genes, a robust phylogenomic framework is indispensable. In the context of the nematode family Diplogastridae to which *P. pacificus* belongs, the identity of a sister group has long been debated. In this work, we generated a pseudochromosome level genome assembly of the nematode *Rhabditoides inermis*, which has previously been proposed as the sister taxon. The genome was assembled from a complex microbial community that is stably associated with *R. inermis* isolates and that consists of multiple bacteria and a fungus, which we identified as a strain of *Vanrija albida*. The *R. inermis* genome spans 173.5Mb that are largely assembled into five pseudochromosomes. This chromosomal configuration likely arose from two recent fusions of different Nigon elements. Phylogenomic analysis did not support a sister group relationship between *R. inermis* and diplogastrids, but rather supports a sister group relationship between the monophyletic Diplogastridae and a group of genera of Rhabditidae including *C. elegans* and *R. inermis*. Thus, our work addresses for the first time the long lasting question about the sister group to diplogastrids at the phylogenomic level and provides with the genomes of *R. inermis* and the associated fungus *V. albida* valuable resources for future genomic comparisons.

**Significance**

Genetic screens are powerful approaches to link phenotypes to genotypes. To trace the evolution of the identified genes and associated traits, a robust phylogenomic context is indispensable. Here, we sequenced the genome of the nematode *Rhabditoides inermis* as previous phylogenetic studies proposed it as sister group to the family Diplogastridae to which the model organism *Pristionchus pacificus* belongs. However, phylogenomic analysis of 40 nematode species did not support this relationship. While this phylogenetic position diminishes the importance of *R. inermis* for our own research, its genome will still be a useful resource for evolutionary genomic studies in nematodes. In addition, our study yielded a high quality genome of the fungus *Vanrija albida* for which no whole genome sequencing data was available.

## Introduction

Nematodes are the most abundant animals on Earth and have colonized virtually all ecosystems including multiple animal and plant hosts (Blaxter et al. 1998; van den Hoogen et al. 2019; Shih et al. 2019). In addition, with *Caenorhabditis elegans* being a nematode, a vast portion of our biological knowledge originates from basic nematode research. To study the evolution of biological processes known from *C. elegans* in other animals, the free-living nematode *P. pacificus* had been introduced as a satellite model organism for comparative biology (Sommer et al. 1996). Recently, new research directions emerged that are unrelated to previous *C. elegans* research. These focused on an evolutionary novelty of the family Diplogastridae to which *P. pacificus* belongs. Specifically, unlike *C. elegans* and many other bacterial-feeding nematodes, *P. pacificus* can form teeth-like denticles that allow killing and predation (Sommer et al. 1996). This predatory form is one of two alternative mouth forms, an example of developmental plasticity (Bento et al. 2010). This research on the regulation of developmental plasticity and predatory feeding behaviors has established *P. pacificus* as a completely independent model system (Ragsdale et al. 2013; Lightfoot et al. 2019).

The family Diplogastridae is a monophyletic taxon that has been subject to intense investigations in the last decade (reviewed in Kanzaki et al. 2014a; Hodda 2022; Mahboob and Tahseen 2024). This included the isolation and characterization of six new diplogastrid genera since 2011 (von Lieven et al. 2011; Kanzaki et al. 2012; Herrmann et al. 2013; Kanzaki et al. 2014b; Ragsdale et al. 2014; Kanzaki et al. 2023). This suggests that our taxonomic knowledge of Diplogastridae is still incomplete. In addition, the sister group of the Diplogastridae is unknown and different candidates have been proposed. For example, the family Bunonematidae was suggested as a sister group based on morphological comparisons (von Lieven 2002) and small subunit ribosomal RNA sequencing (Meldal et al. 2007). In contrast, other rDNA analyses suggested that *Rhabditoides inermis* represents the sister taxon (Kiontke et al. 2007; van Megen et al. 2009; Sudhaus 2023). Recent phylogenomic analysis of the phylum Nematoda did not include *R. inermis* because no genome is available (Ahmed et al. 2022). To fill the existing knowledge gap, we sequenced the *R. inermis* genome.

## Results and Discussion

### Genome Sequencing Data of *R. inermis* Represents a Complex Microbial Community

We generated genomic DNA libraries from the inbred strain RS5678B and sequenced them on the Pacific Biosciences platform. This resulted in 35 Gb of HiFi reads with a median read length of 12 kb. This was assembled into a raw assembly of 2,228 contigs spanning 419 Mb (N50 = 0.4 Mb). To reduce the degree of allelism resulting from remaining heterozygosity in the assembly, we applied the HaploMerger2 software (Huang et al. 2017). This reduced the number of contigs to 782 spanning 253 Mb with an N50 of 0.9 Mb. We further generated Hi-C data and ran the YaHS software to scaffold the *R. inermis* genome (Zhou et al. 2023). Visualization of the average GC content and Hi-C coverage for the resulting scaffolds identified five large scaffolds (>10 Mb) with a GC content of 47% and a high coverage (supplementary figure S1, Supplementary Material online). However, a subset of smaller scaffolds also showed an unusually high or low GC content combined with low coverage. The Hi-C contact map of the largest scaffolds suggested spatial proximity between the five largest scaffolds, but almost no Hi-C signal between the smaller scaffolds (supplementary figure S1, Supplementary Material online). Hypothesizing that the smaller scaffolds could possibly represent microbial contamination, we performed homology searches against the NCBI nr database. This confirmed the microbial origin of these scaffolds (supplementary figure S1, Supplementary Material online). Additional sequencing data from two independently isolated *R. inermis* strains showed some sequencing coverage for all identified microbes, suggesting a stable association between *R. inermis* and its microbial community (supplementary figure S2, Supplementary Material online).

### A Complete Fungal Genome was Assembled From Sequencing Data of *R. inermis* Cultures

While bacterial scaffolds showed more than 90% sequence identity with their best hits in the NCBI database, multiple scaffolds showed lower identity (∼80%) to sequences from the fungal genus *Vanrija* (supplementary figure S1, Supplementary Material online) (Imanishi et al. 2018; Meng et al. 2023). The eight scaffolds comprise 22.5 Mb of genomic sequence with a GC content of around 65%, which is very comparable to genomic data of other *Vanrija* genomes (Imanishi et al. 2018; Meng et al. 2023). Furthermore, benchmarking of universal single copy orthologs (BUSCO) identified 94.8% of the fungal orthologs (Simão et al. 2015; Zdobnov et al. 2017). Phylogenetic analysis of the large subunit (LSU) rRNA and internal transcribed spacer (ITS) sequences from the new assembly together with publicly available sequences from diverse *Vanrija* species on NCBI (supplementary figure S3, Supplementary Material online) grouped our sequences together with sequences from *V. albida*. Finally, to further support that our genome most likely represents *V. albida,* we downloaded 56 *V. albida* sequences from NCBI and performed a BLASTN search against our assembly. This showed a median of 100% identity strongly supporting that our genome assembly represents an isolate of *V. albida*.

### *R. inermis* has Five Chromosomes with Near-Complete Coverage by the Five Largest Scaffolds

**Fig. 1.**
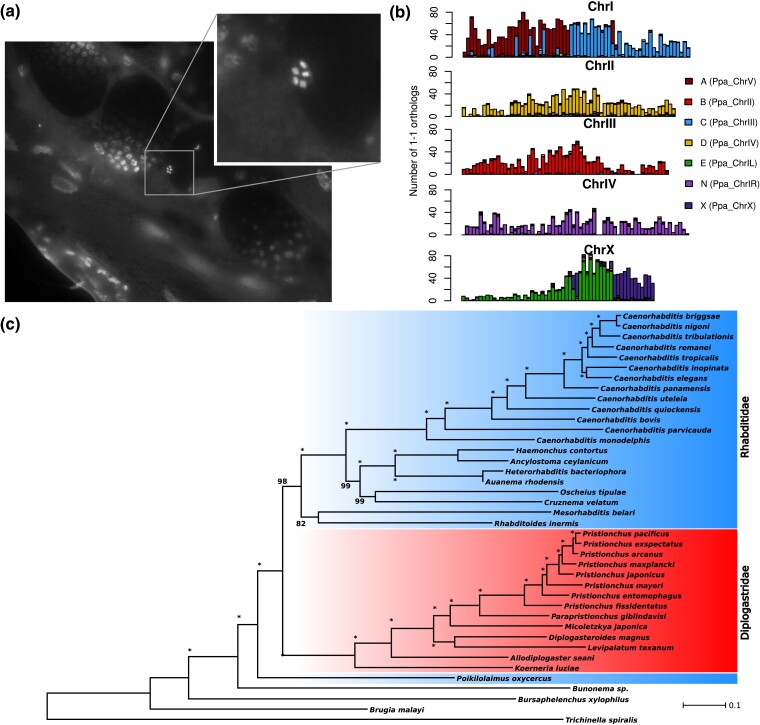
a) Karyotyping of *R. inermis* worms identified oocytes with five chromosomes. b) The bars represent 500-kb windows along the five pseudochromosomes that show the distribution of 1-1 orthologs color-coded by their chromosomal location in the *P. pacificus* genome, which can be used as a proxy to indicate the Nigon elements. c) Phylogenomic and transcriptomic data of diplogastrid and rhabditid nematodes were used to reconstruct a maximum-likelihood tree for the suborder Rhabditina. Note that the family Rhabditidae is known to be paraphyletic (Meldal et al. 2007). The species *B. xylophilus*, *B. malayi*, and *T. spiralis* were included as outgroups. Stars indicate a bootstrap support of 100/100.

To compile a clean version of the *R. inermis* genome, we manually inspected the Hi-C data, GC content, coverage data, and BLASTN results. This yielded a classification into 213 *R. inermis* scaffolds. The completeness of the *R. inermis* genome was estimated by BUSCO to be 88.8% (Table 1) (Simão et al. 2015; Zdobnov et al. 2017). A recent evaluation of 54 nematode genomes yielded a median single-copy completeness value of 85% (Prabh and Rödelsperger 2022) indicating that the *R. inermis* genome is comparable to many other nematodes. A screen for the integrity of telomeres could only identify a large number of telomeric repeats (TTAGGC) at one end of chromosome II indicating that the quality of the genome can be further improved. The final *R. inermis* assembly spans 173.5 Mb with an N50 value of 31.4 Mb (Table 1). The five largest scaffolds comprise 156.7 Mb and thus represent more than 90% of the total assembly suggesting that *R. inermis* has five chromosomes. To obtain further support for this, we performed a Hoechst staining of the gonads as described previously (Wighard et al. 2022). This indeed revealed additional evidence for the presence of five chromosomes in *R. inermis* (Fig. 1a).

**Table 1:** Genome assembly and annotation statistics for the two genomes reported in this study

Species (Taxonomic group)	*R. inermis* (Nematode)	*Vanrija albida* (Fungus)
Assembly size (Mb)	173.5	22.5
Number of contigs/scaffolds	213	8
N50 (Mb)	31.4	3.5
BUSCO genome completeness (%) (single-copy/duplicated/fragmented/missing)	88.8 (83.9/4.9/6.4/4.8)	94.8 (94.8/0.0/1.7/3.5)
Number of genes	23,442	10,349
BUSCO protein completeness (%) (single-copy/duplicated/fragmented/missing)	85.9 (77.3/8.6/7.2/6.9)	91.7(90.0/1.7/6.9/1.4)
Accession	GCA_963966185.1	GCA_037575445.1

The relatively high duplication rate of the *R. inermis* genome (4.9% to 8.6%) suggests that allelic haplotypes could not be completely resolved during the assembly process.

### Nigon Elements are Largely Intact in the *R. inermis* Genome

The structure of most nematode genomes with chromosome-scale assemblies reflects a specific configuration of seven ancestral linkage blocks that had been called Nigon elements A, B, C, D, E, N, and X (Tandonnet et al. 2019; Rödelsperger 2024). These Nigon elements constitute large blocks of macrosynteny between nematode genomes. This means that genes within a Nigon element are frequently reshuffled, whereas rearrangements across different elements are rare. Most nematode chromosomes, specifically in the suborder Rhabditina, correspond to exactly one Nigon element, whereas others are fusions between different Nigon elements (Rödelsperger 2024). The observation of five chromosomes in *R. inermis* suggests that two fusions of Nigon elements occurred. We generated evidence-based gene annotations based on protein homology and transcriptome data with a BUSCO completeness of 86% (Table 1). We used these protein annotations to visualize Nigon elements across the *R. inermis* genome, which indeed revealed two Nigon fusions resulting in *R. inermis* chromosome I and X (Fig. 1b).

### Phylogenomic Analysis Does not Support *R. inermis* as a Sister Taxon to Diplogastridae

To resolve the phylogenetic relationship between *R. inermis* and diplogastrid nematodes, we compiled a phylogenomic data set of 40 species (supplementary table S1, Supplementary Material online) and employed a previously established phylogenomic pipeline for reconstructing a species tree from this data (Rödelsperger et al. 2018). This phylogeny did not support the position of *R. inermis* as sister group to the diplogastrids (Fig. 1c). Importantly, the genus *Poikilolaimus* and *Bunonema* are excluded from this clade and appear as next outgroups. In order to explore potential reasons for the misplacement of *R. inermis* in previous phylogenies, we reanalyzed available nematode sequences for the RNApolII and the 18S and 28S ribosomal RNA markers. Of all three markers, only the 18S sequences supported a sister group relationship (supplementary figure S4, Supplementary Material online). This suggests that the 18S sequence largely contributed to the previous misplacement of *R. inermis*.

## Materials and Methods

### Nematode Culturing and Preparation of Sequencing Libraries

This study used three independent *R. inermis* isolates. The reference strain RS5678B is a derivative of a strain originally obtained from Rebecca Kilner (University of Cambridge) in 2012. This strain was inbred for ten generations to obtain RS5678B. Two other strains (RS6171 and RS6371) were independently isolated in southern Germany. The strain RS5678B was grown on standard nematode growth medium (Pires-daSilva 2013). Worms were washed off of 50 fully grown plates using M9 buffer and then gently pelleted by centrifugation (1,300*×g*, 1 min). Pelleted worms were frozen in liquid nitrogen, ground to a fine powder using a mortar and pestle, and directly transferred into the lysis buffer (QIAGEN genomic DNA extraction kit and genomic tip columns (500/G)). The protocol was performed following the manufacturer's instructions. All steps involving sample vortexing were replaced by sample inverting to limit unwanted DNA shearing. DNA quality and quantity were determined with a NanoDrop ND 1,000 spectrometer (PeqLab, Erlangen, Germany), a Qubit 2.0 Fluorometer (ThermoFisher Scientific, Waltham, USA), and by a Femto pulse system (Agilent, CA, USA). A total of 10 µg genomic DNA was sheared using a Megaruptor 2 device (Diagenode, Denville, USA). Small fragments were removed by a BluePippin size-selection system according to the manufacturer's protocol (P/N 100-286-000-07, Pacific Biosciences, USA). The final library was sequenced on multiplexed SMRT cells of a Pacific Biosciences Sequel II instrument. For Hi-C analysis, *R. inermis* nematodes were grown on standard nematode growth medium and washed off with M9 Buffer. 50 µl frozen worm pellet was ground with a motor in an Eppendorf tube and used for Hi-C Library preparation following the manufacturer's instructions for small animals (Arima Genomics, California, USA). RNA-seq libraries were prepared from mixed-stage cultures as described previously (Rödelsperger et al. 2018). Illumina libraries for the genomes of the strains RS6171 and RS6371 were generated and sequenced by the company Novogene.

### Genome Assembly

Raw PacBio reads were converted into Hifi reads by the ccs program (version 6.4.0) with –min-rq 0.99 option and then demultiplexed by the lima program (version 2.6.0). The Hifi reads were then assembled by the Canu program (version v1.4) with options: -pacbio-corrected -genomeSize = 400 m (Koren et al. 2017). Haplotypes were merged by the program Haplomerger2 (version 20180603) after softmasking repeats in the draft assembly with the help of the Red software (version 05/22/2015) (Huang et al. 2012; Girgis 2015). Hi-C data were aligned against the haplomerged assembly by BWA mem (version 0.7.17-r1188) and scaffolded using the yahs tool (version 1.2a.2) (Zhou et al. 2023; Li and Durbin 2010). For metagenomic classification, we annotated scaffolds with Prokka (version 1.14.6) and performed DIAMOND (version 2.1.4.158) searches against the NCBI NR database (downloaded August 2023) (Seemann 2014; Buchfink et al. 2021). The taxonomic distributions of the best hits were summarized using the taxonomizer library in R. Based on manual inspection of various data sets; we removed all but 213 *R. inermis* scaffolds. At this step, we relabeled the five largest scaffolds as pseudochromosomes (ChrI–ChrX) and manually extracted some regions from scaffolds 1 and 4 that showed some Hi-C signal and merged them as a separate scaffold (ChrUn). Karyotyping analysis was performed as described previously (Wighard et al. 2022).

### Gene Annotation

A de novo transcriptome assembly was assembled from mixed-stage RNA-seq data using trinity (version 2.2.0) with the normalize_reads option (Grabherr et al. 2011). This transcriptome assembly and the current *P. pacificus* gene annotations (version El Paco gene annotations 3) (Athanasouli et al. 2020) were used to generate a set of evidence-based gene annotations using the PPCAC pipeline (Rödelsperger 2021). We then applied a filtering step to remove spurious annotations that likely result from falsely called ORFs in our transcriptome data. Specifically, gene models without homologs in other nematodes (BLASTP e-value < 0.001) were removed if they overlapped conserved genes in antisense direction (Athanasouli et al. 2020; Prabh and Rödelsperger 2022). This resulted in 23,442 gene models with a BUSCO (version 3) completeness score of 85.9% (Table 1, odb9 nematode data set) (Simão et al. 2015; Zdobnov et al. 2017). Application of the PPCAC pipeline to annotate the *Vanrija* assembly yielded 10,349 gene models with 90,1% BUSCO completeness (Table 1, odb9 fungi data set). For coloring the Nigon elements, best reciprocal BLAST hits were computed by the get_BRH.pl of the PPCAC pipeline using the pairwise BLAST output files between *R. inermis* and *P. pacificus* (e-value <0.001).

### Phylogenomic Species Tree

For most species, we obtained protein-coding gene predictions from WormBase ParaSite (supplementary table S1, Supplementary Material online) (Howe et al. 2017). For genes, with multiple annotated isoforms, we used the longest protein as a representative sequence. For other species, we generate protein predictions from available transcriptome data. In such cases, either transcriptome assemblies were downloaded from the European Nucleotide Archive or were generated by the Trinity assembler. Subsequently, the longest partial or complete open reading frame was called per sequence and potential redundant sequences resulting from alternative splicing were removed by running the cd-hit clustering program (version 4.3 with default 90% sequence identity threshold). For the reconstruction of a species tree, we followed a similar procedure as described previously (Rödelsperger et al. 2018).

### Gene Tree Analysis

For each tested marker (ITS and LSU from fungi and RNApolII, 18S, 28S from nematodes), we downloaded corresponding sequences for representative species from NCBI and generated a multiple sequence alignment with MUSCLE (version 3.8.1551) (Edgar 2004). Phylogenetic trees were calculated using the R phangorn package (Schliep 2011). Specifically, the modelTest function was run and a maximum likelihood tree was calculated using bootstrap.pml function selecting the best model based on the Bayes information criterion.

## Supplementary Material

evae230_Supplementary_Data

## Data Availability

All sequencing reads have been uploaded to the European Nucleotide archive under the study accession PRJEB71643. The *Rhabditoides inermis* and the *Vanrija albida* genomes have been deposited under the accessions GCA_963966185.1 and GCA_037575445.1, respectively. The genome data is also available at pristionchus.org.

## References

[evae230-B1] Ahmed M, Roberts NG, Adediran F, Smythe AB, Kocot KM, Holovachov O. Phylogenomic analysis of the phylum Nematoda: conflicts and congruences with morphology, 18S rRNA, and mitogenomes. Front Ecol Evol. 2022:9:769565. 10.3389/fevo.2021.769565.

[evae230-B2] Athanasouli M, Witte H, Weiler C, Loschko T, Eberhardt G, Sommer RJ, Rödelsperger C. Comparative genomics and community curation further improve gene annotations in the nematode *Pristionchus pacificus*. BMC Genomics. 2020:21(1):708. 10.1186/s12864-020-07100-0. 33045985 PMC7552371

[evae230-B3] Bento G, Ogawa A, Sommer RJ. Co-option of the hormone-signalling module dafachronic acid-DAF-12 in nematode evolution. Nature. 2010:466(7305):494–497. 10.1038/nature09164. 20592728

[evae230-B4] Blaxter ML, Ley D, Garey P, Liu JR, Scheldeman LX, Vierstraete P, Vanfleteren A, Mackey JR, Dorris LY, Frisse M, et al A molecular evolutionary framework for the phylum Nematoda. Nature. 1998:392(6671):71–75. 10.1038/32160. 9510248

[evae230-B5] Buchfink B, Reuter K, Drost H-G. Sensitive protein alignments at tree-of-life scale using DIAMOND. Nat Methods. 2021:18(4):366–368. 10.1038/s41592-021-01101-x. 33828273 PMC8026399

[evae230-B6] Edgar RC . MUSCLE: a multiple sequence alignment method with reduced time and space complexity. BMC Bioinformatics. 2004:5(1):113. 10.1186/1471-2105-5-113. 15318951 PMC517706

[evae230-B7] Girgis HZ . Red: an intelligent, rapid, accurate tool for detecting repeats de-novo on the genomic scale. BMC Bioinformatics. 2015:16(1):227. 10.1186/s12859-015-0654-5. 26206263 PMC4513396

[evae230-B8] Grabherr MG, Haas BJ, Yassour M, Levin JZ, Thompson DA, Amit I, Adiconis X, Fan L, Raychowdhury R, Zeng Q, et al Full-length transcriptome assembly from RNA-Seq data without a reference genome. Nat Biotechnol. 2011:29(7):644–652. 10.1038/nbt.1883. 21572440 PMC3571712

[evae230-B9] Herrmann M, Ragsdale EJ, Kanzaki N, Sommer RJ. Sudhausia aristotokia n. gen., n. sp. and S. crassa n. gen., n. sp. (Nematoda: Diplogastridae): viviparous new species with precocious gonad development. Nematology. 2013:15(8):1001–1020. 10.1163/15685411-00002738.

[evae230-B10] Hodda M . Phylum Nematoda: a classification, catalogue and index of valid genera, with a census of valid species. Zootaxa. 2022:5114(1):1–289. 10.11646/zootaxa.5114.1.1. 35391386

[evae230-B11] Howe KL, Bolt BJ, Shafie M, Kersey P, Berriman M. WormBase ParaSite-a comprehensive resource for helminth genomics. Mol Biochem Parasitol. 2017:215:2–10. 10.1016/j.molbiopara.2016.11.005. 27899279 PMC5486357

[evae230-B12] Huang S, Chen Z, Huang G, Yu T, Yang P, Li J, Fu Y, Yuan S, Chen S, Xu A. HaploMerger: reconstructing allelic relationships for polymorphic diploid genome assemblies. Genome Res. 2012:22(8):1581–1588. 10.1101/gr.133652.111. 22555592 PMC3409271

[evae230-B13] Huang S, Kang M, Xu A. HaploMerger2: rebuilding both haploid sub-assemblies from high-heterozygosity diploid genome assembly. Bioinformatics. 2017:33(16):2577–2579. 10.1093/bioinformatics/btx220. 28407147 PMC5870766

[evae230-B14] Imanishi D, Abe K, Kera Y, Takahashi S. Draft genome sequence of the yeast *Vanrija humicola* (formerly cryptococcus humicola) strain UJ1, a producer of d-aspartate oxidase. Genome Announc. 2018:6(11):e00068–e00018. 10.1128/genomeA.00068-18. 29545290 PMC5854778

[evae230-B15] Kanzaki N, Ikeda Y, Shinya R. Onthodiplogaster japonica n. gen., n. sp. (Rhabditida: Diplogastridae) isolated from onthophagus sp. (Coleoptera: Scarabaeidae) from Japan. Sci Rep. 2023:13(1):6470. 10.1038/s41598-023-33586-1. 37081071 PMC10119125

[evae230-B16] Kanzaki N, Ragsdale EJ, Giblin-Davis RM. Revision of the paraphyletic genus Koerneria Meyl, 1960 and resurrection of two other genera of diplogastridae (Nematoda). Zookeys. 2014a:(422):17–30. 10.3897/zookeys.442.7459. PMC4205494 25349487

[evae230-B17] Kanzaki N, Ragsdale EJ, Herrmann M, Mayer WE, Tanaka R, Sommer RJ. Parapristionchus giblindavisi n. gen., n. sp. (Rhabditida: Diplogastridae) isolated from stag beetles (Coleoptera: Lucanidae) in Japan. Nematology. 2012:14(8):933–947. 10.1163/156854112X635878.

[evae230-B18] Kanzaki N, Ragsdale EJ, Susoy V, Sommer RJ. Leptojacobus dorci n. gen., n. sp. (Nematoda: Diplogastridae), an associate of dorcus stag beetles (Coleoptera: Lucanidae). J Nematol. 2014b:46:50–59. 24644371 PMC3957572

[evae230-B19] Kiontke K, Barrière A, Kolotuev I, Podbilewicz B, Sommer R, Fitch DH, Félix MA. Trends, stasis, and drift in the evolution of nematode vulva development. Curr Biol. 2007:17(22):1925–1937. 10.1016/j.cub.2007.10.061. 18024125

[evae230-B20] Koren S, Walenz BP, Berlin K, Miller JR, Bergman NH, Phillippy AM. Canu: scalable and accurate long-read assembly via adaptive k-mer weighting and repeat separation. Genome Res. 2017:27(5):722–736. 10.1101/gr.215087.116. 28298431 PMC5411767

[evae230-B21] Li H, Durbin R. Fast and accurate long-read alignment with burrows–wheeler transform. Bioinformatics. 2010:26(5):589–595. 10.1093/bioinformatics/btp698. 20080505 PMC2828108

[evae230-B22] Lightfoot JW, Wilecki M, Rödelsperger C, Moreno E, Susoy V, Witte H, Sommer RJ. Small peptide-mediated self-recognition prevents cannibalism in predatory nematodes. Science. 2019:364(6435):86–89. 10.1126/science.aav9856. 30948551

[evae230-B23] Mahboob M, Tahseen Q. Diversity, prevalence and microhabitat specificity of nematodes (Rhabditidae Örley, 1880 and diplogastridae micoletzky, 1922) associated with insects: an overview. Int J Pest Manage. 2024:70(2):192–233. 10.1080/09670874.2021.1969470.

[evae230-B24] Meldal BH, Debenham NJ, De Ley P, De Ley IT, Vanfleteren JR, Vierstraete AR, Bert W, Borgonie G, Moens T, Tyler PA, et al An improved molecular phylogeny of the Nematoda with special emphasis on marine taxa. Mol Phylogenet Evol. 2007:42(3):622–636. 10.1016/j.ympev.2006.08.025. 17084644

[evae230-B25] Meng L, Liu C, Zhu L, Wang X, Li C. Draft genome sequence of yeast Vanrija sp. strain TS01, isolated from leukemia patient's urine. Microbiol Resour Announc. 2023:12(9):e0015223. 10.1128/MRA.00152-23. 37610212 PMC10508090

[evae230-B26] Pires-daSilva A. 2013. *Pristionchus pacificus* protocols. In: Wormbook. ed. The C. elegans Research Community, WormBook, doi/10.1895/wormbook.1.114.2., http://www.wormbook.org. PMC5402221 23505072

[evae230-B27] Prabh N, Rödelsperger C. Multiple *Pristionchus pacificus* genomes reveal distinct evolutionary dynamics between de novo candidates and duplicated genes. Genome Res. 2022:32(7):1315–1327. 10.1101/gr.276431.121. 35618417 PMC9341508

[evae230-B28] Ragsdale EJ, Kanzaki N, Sommer RJ. 2014. Levipalatum texanum n. gen., n. sp. (Nematoda: Diplogastridae), an androdioecious species from the south-eastern USA. Nematology. 16(6):695–709. 10.1163/15685411-00002798.

[evae230-B29] Ragsdale EJ, Müller MR, Rödelsperger C, Sommer RJ. A developmental switch coupled to the evolution of plasticity acts through a sulfatase. Cell. 2013:155(4):922–933. 10.1016/j.cell.2013.09.054. 24209628

[evae230-B30] Rödelsperger C . The community-curated *Pristionchus pacificus* genome facilitates automated gene annotation improvement in related nematodes. BMC Genomics. 2021:22(1):216. 10.1186/s12864-021-07529-x. 33765927 PMC7992802

[evae230-B31] Rödelsperger C . Comparative genomics of sex, chromosomes, and sex chromosomes in *Caenorhabditis elegans* and other Nematodes. Methods Mol. Biol. 2024:2802:455–472. 10.1007/978-1-0716-3838-5_15. 38819568

[evae230-B32] Rödelsperger C, Röseler W, Prabh N, Yoshida K, Weiler C, Herrmann M, Sommer RJ. Phylotranscriptomics of pristionchus Nematodes reveals parallel gene loss in six hermaphroditic lineages. Curr Biol. 2018:28(19):3123–3127.e5. 10.1016/j.cub.2018.07.041. 30245109

[evae230-B33] Schliep KP . Phangorn: phylogenetic analysis in R. Bioinformatics. 2011:27(4):592–593. 10.1093/bioinformatics/btq706. 21169378 PMC3035803

[evae230-B34] Seemann T . Prokka: rapid prokaryotic genome annotation. Bioinformatics. 2014:30(14):2068–2069. 10.1093/bioinformatics/btu153. 24642063

[evae230-B35] Shih PY, Lee JS, Shinya R, Kanzaki N, Pires-daSilva A, Badroos JM, Goetz E, Sapir A, Sternberg PW. Newly identified Nematodes from mono lake exhibit extreme arsenic resistance. Curr Biol. 2019:29(19):3339–3344.e4. 10.1016/j.cub.2019.08.024. 31564490

[evae230-B36] Simão FA, Waterhouse RM, Ioannidis P, Kriventseva EV, Zdobnov EM. BUSCO: assessing genome assembly and annotation completeness with single-copy orthologs. Bioinformatics. 2015:31(19):3210–3212. 10.1093/bioinformatics/btv351. 26059717

[evae230-B37] Sommer RJ, Carta LK, Kim S-Y, Sternberg PW. Morphological, genetic and molecular description of *Pristionchus pacificus* sp. n.(Nematoda: Neodiplogasteridae). Fundam Appl Nematol. 1996:19:511–522.

[evae230-B38] Sudhaus W . An update of the catalogue of paraphyletic ‘Rhabditidae’ (Nematoda) after eleven years. Soil Org. 2023:95:95–116. 10.25674/so95iss1id312.

[evae230-B39] Tandonnet S, Koutsovoulos GD, Adams S, Cloarec D, Parihar M, Blaxter ML, Pires-daSilva A. Chromosome-Wide evolution and sex determination in the three-sexed nematode auanema rhodensis. G3 (Bethesda). 2019:9(4):1211–1230. 10.1534/g3.119.0011. 30770412 PMC6469403

[evae230-B40] van den Hoogen J, Geisen S, Routh D, Ferris H, Traunspurger W, Wardle DA, de Goede RGM, Adams BJ, Ahmad W, Andriuzzi WS, et al Soil nematode abundance and functional group composition at a global scale. Nature. 2019:572(7768):194–198. 10.1038/s41586-019-1418-6. 31341281

[evae230-B41] van Megen H, van den Elsen S, Holterman M, Karssen G, Mooyman P, Bongers T, Holovachov O, Bakker J, Helder J. A phylogenetic tree of nematodes based on about 1200 full-length small subunit ribosomal DNA sequences. Nematology. 2009:11(6):927–950. 10.1163/156854109X456862.

[evae230-B42] von Lieven AF . The sister group of the diplogastrina (Nematoda). Russ J Nematol. 2002:10:127–138.

[evae230-B43] von Lieven AF, Uni S, Ueda K, Barbuto M, Bain O. Cutidiplogaster manati n. gen., n. sp. (Nematoda: Diplogastridae) from skin lesions of a west Indian manatee (sirenia) from the okinawa churaumi aquarium. Nematology. 2011:13(1):51–59. 10.1163/138855410X500082.

[evae230-B44] Wighard SS, Athanasouli M, Witte H, Rödelsperger C, Sommer RJ. A new hope: a hermaphroditic nematode enables analysis of a recent whole genome duplication event. Genome Biol Evol. 2022:14(12):evac169. 10.1093/gbe/evac169. 36461901 PMC9763058

[evae230-B45] Zdobnov EM, Tegenfeldt F, Kuznetsov D, Waterhouse RM, Simão FA, Ioannidis P, Seppey M, Loetscher A, Kriventseva EV. OrthoDB v9.1: cataloging evolutionary and functional annotations for animal, fungal, plant, archaeal, bacterial and viral orthologs. Nucleic Acids Res. 2017:45(D1):D744–D749. 10.1093/nar/gkw1119. 27899580 PMC5210582

[evae230-B46] Zhou C, McCarthy SA, Durbin R. YaHS: yet another Hi-C scaffolding tool. Bioinformatics. 2023:39(1):btac808. 10.1093/bioinformatics/btac808. 36525368 PMC9848053

