# Supplementary material for: Genome Assembly of the Nematode Rhabditoides Inermis From a Complex Microbial Community
Source: Genome Biol Evol. 2024 Nov 7;16(11):evae230. doi: 10.1093/gbe/evae230 (PMC11542624; doi:10.1093/gbe/evae230)
Supplement: evae230_Supplementary_Data [file evae230_supplementary_data.pdf]

Supplementary Material to

Genome assembly of the nematode *Rhabditoides inermis* from a complex microbial community

Christian Rödelsperger[1,*], Waltraud Röseler[1], Marina Athanasouli[1], Sara Wighard[1,2], Matthias Herrmann[1], Ralf J. Sommer[1,*]

[1] Department for Integrative Evolutionary Biology, Max Planck Institute for Biology, Max-Planck-Ring 9, 72076 Tübingen, Germany

[2] Current address: Institute of Molecular Biotechnology of the Austrian Academy of Sciences (IMBA), Vienna BioCenter (VBC), 1030 Vienna, Austria.

[*] Corresponding authors:

christian.roedelsperger@tuebingen.mpg.de

ralf.sommer@tuebingen.mpg.de

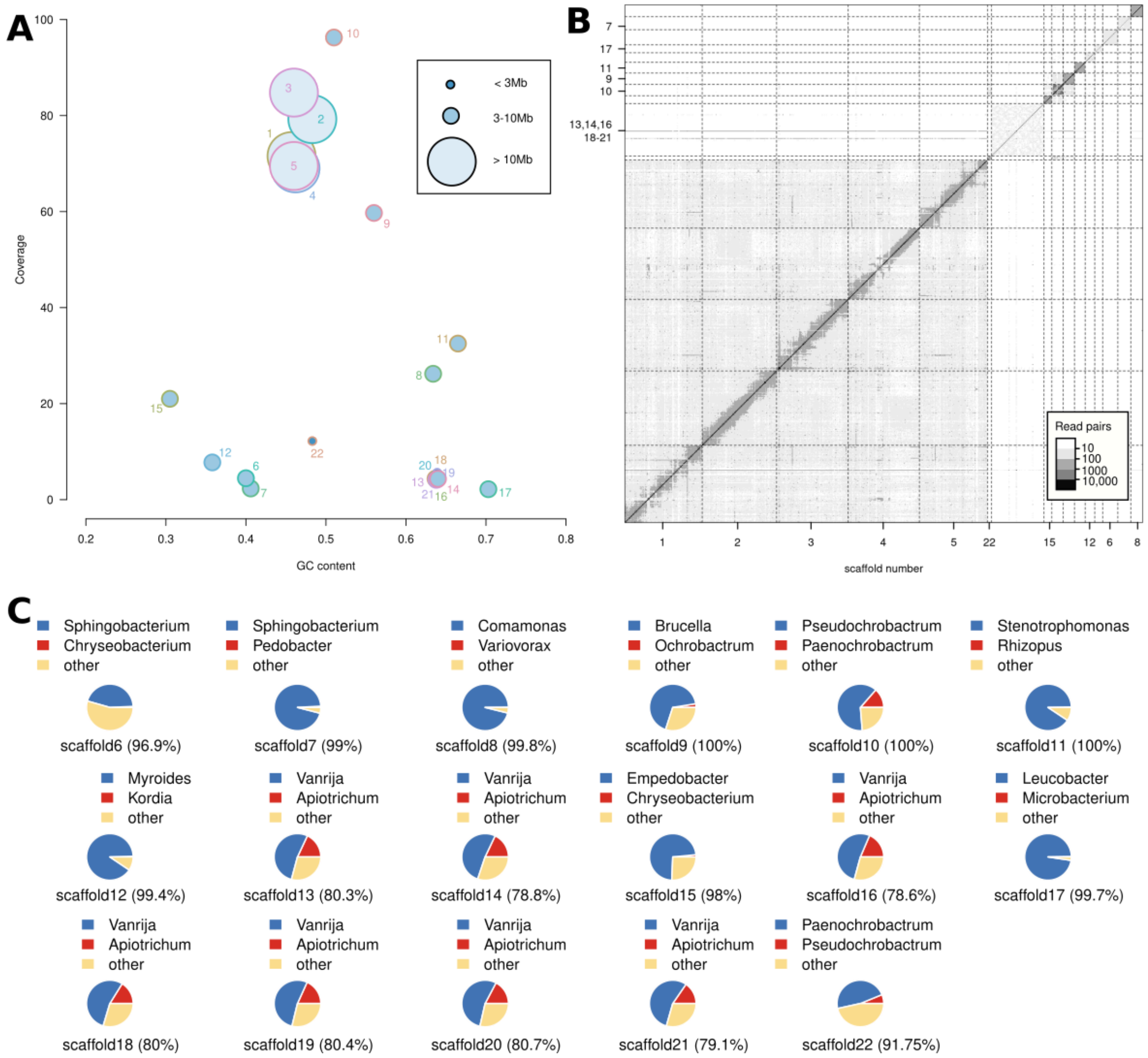

Fig. S1. Genome assembly of a complex microbial community. A Visualization of GC content and Hi-C coverage indicates that the five largest scaffolds appear to be different from the smaller ones. **B** The Hi-C contact map of the 22 largest scaffolds shows a background level of Hi-C signal across the five largest scaffolds but not between the smaller scaffolds. **C** For scaffolds 6-22 we performed homology searches against the NCBI nr database. The pie charts summarize the taxonomic distribution for the best hits at the genus level.

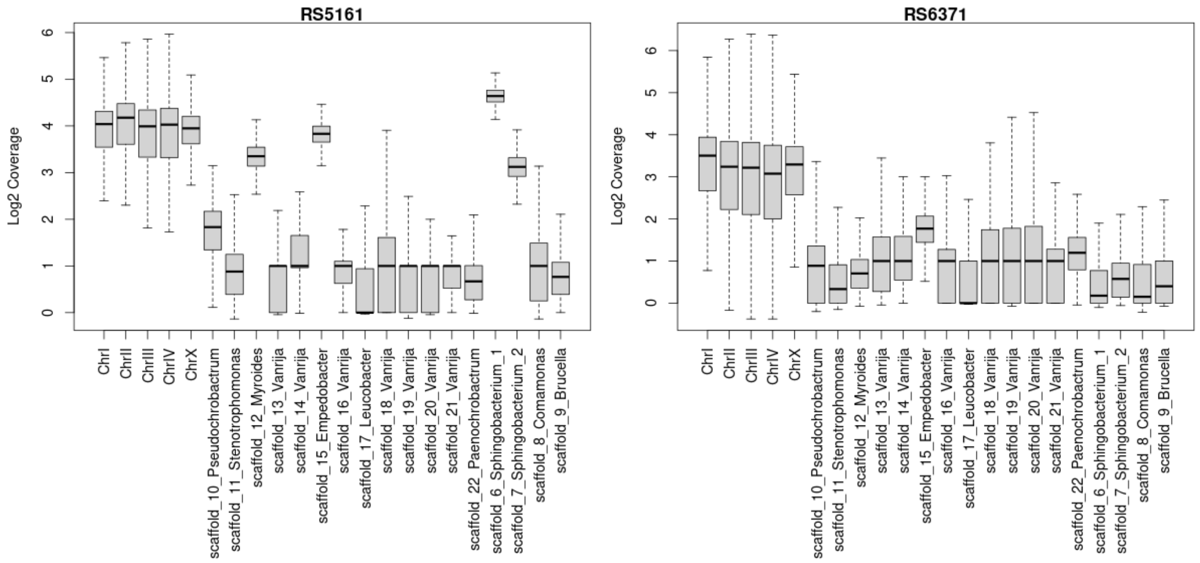

Fig. S2. Coverage of microbial scaffolds in different *R. inermis* isolates. Coverage analysis reveals evidence for the presence of all microbes in sequencing data of two independent isolates of *R. inermis*. The y-axis indicates the average Log2 coverage in 1-kb windows for the largest scaffolds/chromosomes.

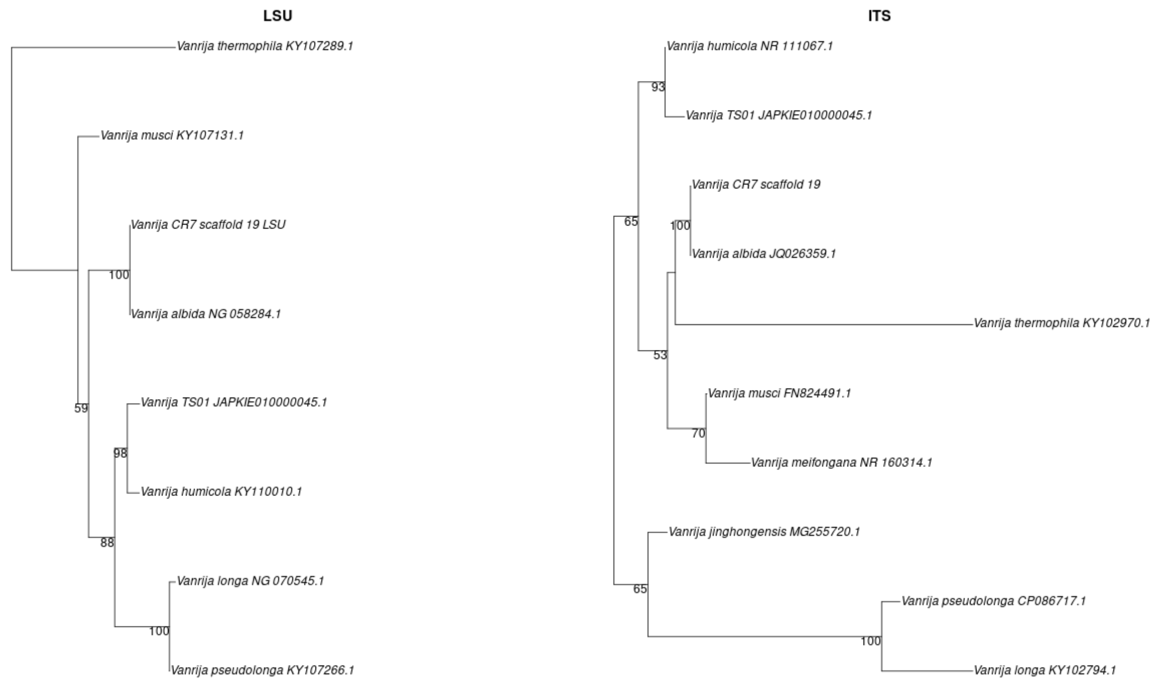

Fig. S3. Phylogenetic relationships between different *Vanrija* species. LSU and ITS sequences from different *Vanrija* species were downloaded from NCBI Genbank, aligned with MUSCLE, and maximum likelihood trees were calculated by the phangorn R package. For both markers, sequences from our genome assembly (CR7) group together with sequences from *V. albida*.

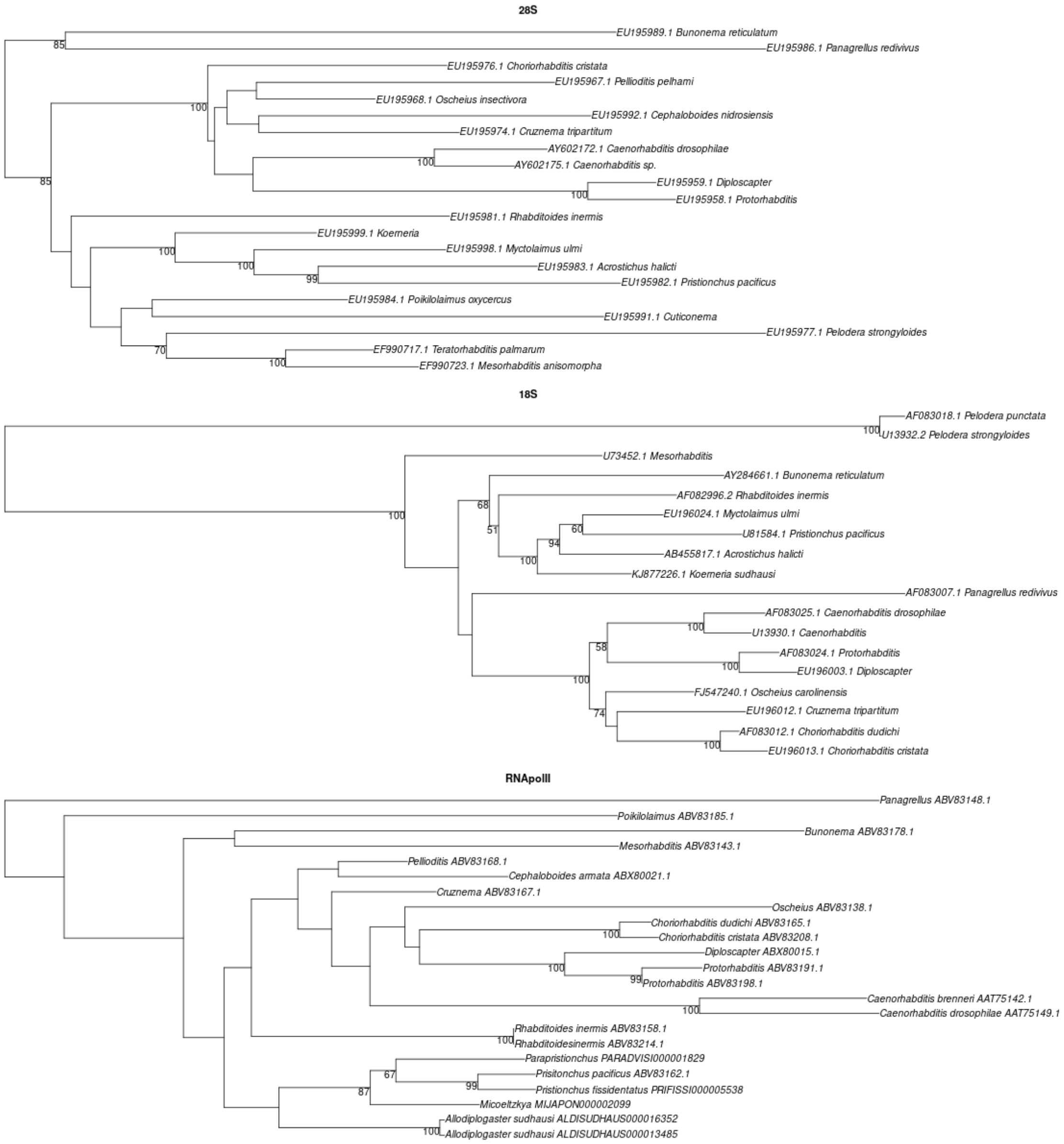

Fig. S4. Phylogenetic relationships between different nematodes. Phylogenetic trees were reconstructed for RNApolII, 18S and 28S ribosomal RNAs from different nematodes. Only the 18S data suggests a sister group relationship between R. inermis and the family Diplogastridae.

Table S1 - Nematode genomic and transcriptomic data sets

Species	Source accession / version	Type	Reference
Allodiplogaster seani	European Nucleotide Archive: HCAZ01000000	RNA	(Wighard et al. 2022)

Ancylostoma ceylanicum	WormBase ParaSite WBPS18 PRJNA231479	Protein	(Schwarz et al. 2015)
Auanema rhodensis	European Nucleotide Archive: ERR3150287	RNA	(Tandonnet et al. 2019)
Bunoema sp.	European Nucleotide Archive: GITZ01000000	RNA	(Casasa et al. 2021)
Brugia malayi	WormBase ParaSite WBPS14 PRJNA10729	Protein	(Ghedin et al. 2007)
Bursaphelenchus xlyophilus	WormBase ParaSite WBPS14 PRJEA64437	Protein	(Kikuchi et al. 2011)
Caenorhabditis bovis	WormBase ParaSite WBPS18 PRJEB34497	Protein	(Stevens et al. 2019)
Caenorhabditis briggsae	WormBase ParaSite WBPS14 PRJNA10731	Protein	-
Caenorhabditis elegans	WormBase ParaSite WBPS14 PRJNA13758	Protein	-
Caenorhabditis inopinata	WormBase ParaSite WBPS18 PRJDB5687	Protein	(Kanzaki et al. 2018)
Caenorhabditis monodelphis	caenorhabditis.org	Protein	(Slos et al. 2017)
Caenorhabditis nigoni	WormBase ParaSite WBPS18 PRJNA384657	Protein	(Yin et al. 2018)
Caenorhabditis panamensis	WormBase ParaSite WBPS18 PRJEB28259	Protein	(Stevens et al. 2019)
Caenorhabditis parvicauda	WormBase ParaSite WBPS18 PRJEB12595	Protein	(Stevens et al. 2019)
Caenorhabditis quiockensis	WormBase ParaSite WBPS18 PRJEB11354	Protein	(Stevens et al. 2019)
Caenorhabditis remanei	WormBase ParaSite WBPS18 PRJNA248911	Protein	(Fierst et al. 2015)
Caenorhabditis tribulationis	WormBase ParaSite WBPS18 PRJEB12608	Protein	(Stevens et al. 2019)
Caenorhabditis tropicalis	WormBase ParaSite WBPS18 PRJNA53597	Protein	-
Caenorhabditis uteleia	WormBase ParaSite WBPS18 PRJEB12600	Protein	(Stevens et al. 2019)
Cruznema velatum	European Nucleotide Archive: SRR23934717	RNA	(Guo et al. 2023)
Diplogasteroides magnus	European Nucleotide Archive: GITX01000000	RNA	(Casasa et al. 2021)

Haemonchus contortus	WormBase ParaSite WBPS14 PRJEB506	Protein	(Doyle et al. 2020)
Heterorhabditis bacteriophora	European Nucleotide Archive: SRR6294669	RNA	(Vadnal et al. 2018)
Koerneria luziae	European Nucleotide Archive: GIUA01000000	RNA	(Casasa et al. 2021)
Levipalatum texanum	European Nucleotide Archive: GITY01000000	RNA	(Casasa et al. 2021)
Micoletzkya japonica	pristionchus.org	Protein	(Prabh et al. 2018)
Oscheius tipulae	WormBase ParaSite WBPS14 PRJEB15512	Protein	(Besnard et al. 2017)
Poikilolaimus oxycercus	European Nucleotide Archive: SRR6049087	Protein	(Beltran et al. 2019)
Pristionchus arcanus	pristionchus.org PPCAC (version 1)	Protein	(Prabh et al. 2018)
Pristionchus entomophagus	pristionchus.org PPCAC (version 1)	Protein	(Prabh et al. 2018)
Pristionchus exspectatus	pristionchus.org Yoshida et al.	Protein	Yoshida et al. 2023
Pristionchus fissidentatus	pristionchus.org PPCAC (version 1)	Protein	(Prabh et al. 2018)
Pristionchus japonicus	pristionchus.org PPCAC (version 1)	Protein	(Prabh et al. 2018)
Pristionchus mayeri	pristionchus.org PPCAC (version 1)	Protein	(Prabh et al. 2018)
Pristionchus maxplancki	pristionchus.org PPCAC (version 1)	Protein	(Prabh et al. 2018)
Pristionchus pacificus	pristionchus.org, El Paco gene annotation 3	Protein	(Athanasouli et al. 2020)
Parapristionchus giblindavisi	pristionchus.org (version 2, 2022)	Protein	(Röseler et al. 2022)
Rhabditoides inermis	this study	Protein	this study
Trichinella spiralis	WormBase ParaSite WBPS14 PRJNA12603	Protein	(Mitreva et al. 2011)

References

Athanasouli M et al. 2020. Comparative genomics and community curation further improve gene annotations in the nematode Pristionchus pacificus. BMC Genomics. 21:708.

Beltran T et al. 2019. Comparative Epigenomics Reveals that RNA Polymerase II Pausing

and Chromatin Domain Organization Control Nematode piRNA Biogenesis. Dev. Cell. 48:793–810.e6.

Besnard F, Koutsovoulos G, Dieudonné S, Blaxter M, Félix M-A. 2017. Toward Universal Forward Genetics: Using a Draft Genome Sequence of the Nematode Oscheius tipulae To Identify Mutations Affecting Vulva Development. Genetics. 206:1747–1761.

Casasa S, Biddle JF, Koutsovoulos GD, Ragsdale EJ. 2021. Polyphenism of a Novel Trait Integrated Rapidly Evolving Genes into Ancestrally Plastic Networks. Mol. Biol. Evol. 38:331–343.

Doyle SR et al. 2020. Genomic and transcriptomic variation defines the chromosome-scale assembly of Haemonchus contortus, a model gastrointestinal worm. Commun Biol. 3:656.

Fierst JL et al. 2015. Reproductive Mode and the Evolution of Genome Size and Structure in Caenorhabditis Nematodes. PLoS Genet. 11:e1005323.

Ghedin E et al. 2007. Draft genome of the filarial nematode parasite Brugia malayi. Science. 317:1756–1760.

Guo F et al. 2023. Transcriptomics of Cruznema velatum (Nematoda: Rhabditidae) with a redescription of the species. J. Helminthol. 97:e57.

Kanzaki N et al. 2018. Biology and genome of a newly discovered sibling species of Caenorhabditis elegans. Nat. Commun. 9:3216.

Kikuchi T et al. 2011. Genomic insights into the origin of parasitism in the emerging plant pathogen Bursaphelenchus xylophilus. PLoS Pathog. 7:e1002219.

Mitreva M et al. 2011. The draft genome of the parasitic nematode Trichinella spiralis. Nat. Genet. 43:228–235.

Prabh N et al. 2018. Deep taxon sampling reveals the evolutionary dynamics of novel gene families in Pristionchus nematodes. Genome Res. 28:1664–1674.

Röseler W et al. 2022. The improved genome of the nematode Parapristionchus giblindavisi provides insights into lineage-specific gene family evolution. G3 . 12. doi: 10.1093/g3journal/jkac215.

Schwarz EM et al. 2015. The genome and transcriptome of the zoonotic hookworm Ancylostoma ceylanicum identify infection-specific gene families. Nat. Genet. 47:416–422.

Slos D, Sudhaus W, Stevens L, Bert W, Blaxter M. 2017. Caenorhabditis monodelphis sp. n.: defining the stem morphology and genomics of the genus Caenorhabditis. BMC Zoology. 2:1–15.

Stevens L et al. 2019. Comparative genomics of 10 new Caenorhabditis species. Evol Lett. 3:217–236.

Tandonnet S et al. 2019. Chromosome-Wide Evolution and Sex Determination in the Three-Sexed Nematode Auanema rhodensis. G3 . 9:1211–1230.

Vadnal J et al. 2018. Refined ab initio gene predictions of Heterorhabditis bacteriophora using RNA-seq. Int. J. Parasitol. 48:585–590.

Wighard SS, Athanasouli M, Witte H, Rödelsperger C, Sommer RJ. 2022. A New Hope: A Hermaphroditic Nematode Enables Analysis of a Recent Whole Genome Duplication Event.

Genome Biol. Evol. 14. doi: 10.1093/gbe/evac169.

Yin D et al. 2018. Rapid genome shrinkage in a self-fertile nematode reveals sperm competition proteins. Science. 359:55–61.
